# Purification and characterization of L-arginine deiminase from *Penicillium chrysogenum*

**DOI:** 10.1186/s12866-024-03192-w

**Published:** 2024-01-31

**Authors:** Hamed M. El-Shora, Nessma A. El-Zawawy, Mohamed A. Abd El-Rheem, Metwally A. Metwally

**Affiliations:** 1 Department of Botany, Faculty of Science, Mansoura University, Mansoura, Egypt; 2 Department of Botany, Faculty of Science, Tanta University, Tanta, Egypt

**Keywords:** Arginine deiminase, *P.chrysogenum*, Purification, Kinetics, Activation, Active groups

## Abstract

L-arginine deiminase (ADI, EC 3.5.3.6) hydrolyzes arginine to ammonia and citrulline which is a natural supplement in health care. ADI was purified from *Penicillium chrysogenum* using 85% ammonium sulfate, DEAE-cellulose and Sephadex G_200_. ADI was purified 17.2-fold and 4.6% yield with a specific activity of 50 Umg^− 1^ protein. The molecular weight was 49 kDa. ADI expressed maximum activity at 40^o^C and an optimum pH of 6.0. ADI thermostability was investigated and the values of both t_0.5_ and D were determined. K_d_ increased by temperature and the Z value was 38^o^C. ATP, ADP and AMP activated ADI up to 0.6 mM. Cysteine and dithiothreitol activated ADI up to 60 µmol whereas the activation by thioglycolate and reduced glutathione (GSH) prolonged to 80 µmol. EDTA, α,α-dipyridyl, and *o*-phenanthroline inactivated ADI indicating that ADI is a metalloenzyme. N-ethylmaleimide (NEM), N-bromosuccinimide (NBS), butanedione (BD), dansyl chloride (DC), diethylpyrocarbonate (DEPC) and N-acetyl-imidazole (NAI) inhibited ADI activity indicating the necessity of sulfhydryl, tryptophanyl, arginyl, lysyl, histidyl and tyrosyl groups, respectively for ADI catalysis. The obtained results show that ADI from *P. chrysogenum* could be a potential candidate for industrial and biotechnological applications.

## Introduction

The enzymatic reactions are important for the life of the organisms and enzymes assist life processes in all life forms from viruses to man. These reactions have been naturally designed to function under various physiological conditions [1], for replacement of old tissues [2], conversion of food into energy [3] and disposal of toxic materials [4].

L-arginine deiminase (ADI: EC 3.5.3.6) belongs to the hydrolases and catalyzes the nucleophilic substitution reactions at the guanidinium carbon atom of L-arginine or its derivatives [5]. ADI catalyzes the irreversible conversion of L-arginine to ammonia and L-citrulline [6]. ADI is derived from microorganisms but is not produced by mammals. Since ADI is a foreign protein, multiple injections are needed to produce the desired effect of L-arginine deprivation [7].

ADI is reported in bacteria e.g. *Streptococcus pyogenes* [5, 8, 9] and lactic acid bacteria [10]. Literature on fungal ADIs is scarce as there are only a fee reports including that on *Aspergillus fumigatus* [11] and *Aspergillus nidulans* [12]. Improvement of ADI stability was taken into consideration for use in biosensors. Efforts have been made for enhancing the stability of ADI e.g., pH stability, long-term stability [1] and high-temperature stability [13].

ADI is applied in the production of L-citrulline which is a non-essential amino acid as a natural supplement in health care [14]. Also, L-citrulline can improve sexual functions in both men and women [15].

A biosensor based on L-arginine deiminase was developed for the detection of L-arginine [16, 17]. Arginine deaminase (ADI) is an important anticancer drug worldwide used in the chemotherapy of arginine-auxotrophic tumors, such as hepatocellular carcinomas and melanomas [18]. ADI which degrade arginine, are potential tumor growth inhibitors [19, 20]. L-citrulline is therefore an ideal natural supplement that may help to prevent cancer, stroke, and heart disease.

These multiple functions in the healthcare industry have seen a large growth in the global L-citrulline market. Due to food security, energy and environmental concerns, a simple, safe, energy-efficient method for L-citrulline production is much needed [21]. The present work aimed to isolate, purify and characterize ADI from *P. chrysogenum*.

## Materials and methods

### Experimental microorganism

*Penicillium chrysogenum* Thom AUMC 14,100 gb (Accession No. MN219732) was obtained from Assiut University Mubasher Mycological Center (AUMMC), Assiut-Egypt-71,516.

### Inoculum preparation

Spore suspension of *P. chrysogenum* was prepared by scraping the surface of a 6-day-old sporulating culture in 10 ml of 0.85% sterile saline solution containing 0.1% Tween-80. Each ml of spore suspension which contains 10^7^ spores was counted using a hemocytometer and used as the inoculum.

### Production of ADI

Modified Czapek Dox liquid medium was prepared in 250 ml Erlenmeyer flasks each containing 50 ml (pH 6.0) [22] using 0.7 g 100 ml^− 1^ of glucose as carbon source and 0.5 g 100 ml^− 1^ of yeast extract as nitrogen source. After sterilization, the flasks were inoculated with two ml of spore suspension at a concentration of 10^7^ spores ml^− 1^. The flasks after inoculation were incubated at 35^o^C for 6 days in the dark with an agitation rate of 150 rpm with 0.7 g 100 ml^− 1^ of L-arginine. The cultures were filtrated by Whatman no.1 filter paper. The obtained mycelia were washed twice with potassium phosphate (pH 7.5) and used for enzyme preparation.

### Preparation of ADI from *P. chrysogenum*

The culture supernatant and pellet (mycelia mat) were separated by filtration. The supernatant was discarded and 1 g of the pellet was suspended in 100 ml of 0.2 M sodium acetate buffer, pH 6.0, and homogenized well with hand grinder and kept in an ice bath. The extract was further disrupted by ultrasonification (UltraTurax, T 2.5) at 4^o^C. the disruption process was not continuous, only 5 to 10 s at each time to finally 1 min. From this solution, cell debris was separated by centrifugation at 10,000 rpm for 10 min at 4^o^C and filtered.

### ADI assay

Assay was done as per Choi et al. [23]. The reaction medium contains 500 µl of 150 mM L-arginine in 150 mM phosphate buffer (pH 7.5) and 500 µl of ADI preparation. The reaction was incubated at 40 °C for 30 min, terminated by 10% (w/v) TCA and then centrifuged for 10 min at 6000 g. A sample of 50 µl of the supernatant was fused with 0.2 ml Nessler’s reagent, and the developed color was recorded at 500 nm. One unit of ADI was expressed as the amount of enzyme required to release one µmol of ammonia per min under standard assay conditions [11].

### Purification of ADI from *P. chrysogenum*

The crude extract of ADI was treated with (NH_4_)_2_SO_4_ to precipitate the enzyme by gently stirring in an ice bath, till 85% saturation according to Bollag et al. [24]. The mixture was left at 4^o^C overnight and centrifuged at 5000 rpm for 15 min. The collected pellets were suspended in a phosphate buffer (pH 7.0). The precipitated protein was dialyzed against the same buffer using a dialysis membrane. The dialysate was uploaded onto column of DEAE-cellulose as anion exchange chromatography and the ADI activity as well as the protein content was measured in the eluent. The eluted enzyme was uploaded onto Sephadex G_200_ column. The enzyme was eluted by potassium phosphate buffer (pH 7.0) at 1 ml 10 min^− 1^ flow rate. ADI activity and protein contents of the eluted fractions were determined as mentioned above. The most active fractions were combined to investigate the biochemical characteristics. The protein content was determined by the method of Bradford [25].

### Molecular weight analysis by SDS-PAGE

The homogeneity of purified ADI was confirmed by polyacrylamide gel electrophoresis by Laemmli [26]. The molecular mass of the resulting protein band was calculated from the authentic protein markers were purchased from Sigma-Aldrich.

### Effect of pH, temperature and substrate concentration on ADI activity

The influence of L-arginine on ADI activity was studied using different concentrations (0.2, 0.4, 0.6, 0.8 and 1.0 mM). The reaction mixture was incubated at 40^o^C for 30 min, followed by ADI assay. K_m_ and V_max_ values were calculated from the Eadie-Hofstee plot by plotting V/S against V for the enzyme. The effect of the pH of the reaction medium on ADI activity was estimated at various pH values (3, 4, 5, 6, 7, 8 and 9) using various buffers: 50 mM acetate buffer (pH 3.0–5.0), 50 mM phosphate buffer (pH 5.0–8.0) and 50 mM sodium carbonate buffer (pH 9.0–10.0). ADI activity was estimated at different temperatures (20-50^o^C). The other factors affecting ADI activity including ADI concentration, pH and L-arginine concentration were retained constant.

### Thermostability of purified ADI

The thermostability of the purified ADI was assessed by pre-incubation of ADI without substrate at 50, 55, 60 and 65^o^C. The remaining ADI activity was estimated after 10, 20, 30, 40, 50 and 60 min without any additives. The half-life (t_0.5_) was calculated from a plot of the residual activity vs. the time and the deactivation rate constants (K_d_) were estimated using the equation: t_0.5_ = Ln (2)/ K_d_ [27]. Half-life (t_0.5_) is the required time for a reduction of 50% of the initial ADI activity at a particular temperature [28]. The D value was calculated using the equation: D = Ln (10)/ K_d_ [27]. The D-value (decimal reduction time) is defined as the needed time for a 90% decrease in the original activity and was calculated as described by Singh and Wadhwa [29].

The dependence of the D-value on the temperature is expressed by the Z-value. The Z value is the temperature rise essential for the reduction of the D-value by one logarithmic cycle on the curve and was calculated from the equation: Log (D_2_/ D_1_) = (T_1_-T_2_)/ Z [30]. The Z value was calculated from the slope of the graph between Log D versus T (°C) using the equation: Slope = -1/ Z [27]. The Z-value indicates how many degrees of temperature are required for decimal reduction time to be tenfold higher or lower [28].

### Effect of adenosine and thiol compounds on ADI activity

The effect of adenosine compounds (ATP, ADP and AMP) on ADI activity was tested at various concentrations (0.2, 0.4, 0.6, 0.8 and 1.0 mM) in the reaction medium followed by determination of ADI activity. The effect of sulfur-containing compounds such as cysteine, dithiothreitol (DTT), thioglycolate and glutathione (GSH) was investigated at 20, 40, 60, 80 and 100 µmol in the reaction mixture followed by measuring ADI activity.

### Effect of chelating agents on the activity of purified ADI

The effect of EDTA, α,α-dipyridyl and *o*-phenanthroline on ADI activity was exanimated at 2, 4, 6, 8 and 10 mM in the enzyme reaction mixture followed by determining the enzyme activity.

### Effect of N-ethylmaleimide (NEM)

Detection of the –SH group in purified ADI was carried out according to El-Shora and Metwally [31]. The purified enzyme (2.5 Uml^− 1^) was incubated at 25^o^C for 1 h in 100 mM Tris-HCl (pH 5.0), and 1 mM EDTA with various NEM concentrations (2, 4, 6, 8, and 10 mM). After 1 h of incubation, aliquots were taken and examined for ADI activity.

### Effect of N-bromosuccinimide (NBS)

Detection of the tryptophanyl group was done by the method of [31]. Aliquot from 2, 4, 6, 8, and 10 mM of NBS was added to 10 ml of ADI enzyme in 150 mM phosphate buffer (pH 7.5). Additions were performed slowly and using a magnetic stirrer the formation of turbidity in the solution. After 1 h of stirring, the modified enzyme was dialyzed against distilled water at 28 ± 2^o^C for 24 h and then assayed for ADI activity [32].

### Effect of butanedione (BD)

The ADI modification by BD was carried out at room temperature according to Bihzad and El-Shora [33]. The butanedione-borate solution was tested at different concentrations (2, 4, 6, 8 and 10 mM). Aliquot from each concentration was mixed with 10 ml of ADI in 50 mM sodium borate buffer (pH 7.0) to initiate the modification reaction. ADI activity was determined immediately afterwards.

### Effect of dansyl chloride (DC)

Modification of the lysyl group was carried out according to El-Shora [34]. Stock solutions of DC in acetonitrile were freshly prepared. The enzyme sample (0.5 ml) was incubated at 4^o^C for 1 h with various concentrations of DC (2, 4, 6, 8, and 10 mM) in 100 mM Tris-HCl (pH 7.0). The reaction was terminated by adding 20 mM *β*-mercaptoethanol and 30 mM lysine.

### Effect of diethylpyrocarbonate (DEPC)

Detection of the histidyl group was carried out according to El-Shora and Metwally [31]. The solution of DEPC was freshly prepared. The purified enzyme (10 ml) was combined with 2, 4, 6, 8 and 10 mM DEPC at 25^o^C in the assay buffer for 5 min and subsequently, the activity of ADI was determined.

### Effect of N-acetyl-imidazole (NAI)

The reaction mixture composed of 150 mM phosphate buffer (pH 7.5) containing ADI and 2, 4, 6, 8 and 10 mM NAI, was incubated at 37 °C for 20 min followed by measuring ADI activity [35].

### Statistical analysis

Data are expressed as the mean ± standard deviation (SD) of three replicates. Different letters represent the statistical components between groups by using one-way ANOVA and post hoc Duncan’s test (*p* < 0.05).

## Results

### Purification of ADI from *P. chrysogenum*

**Fig. 1:**
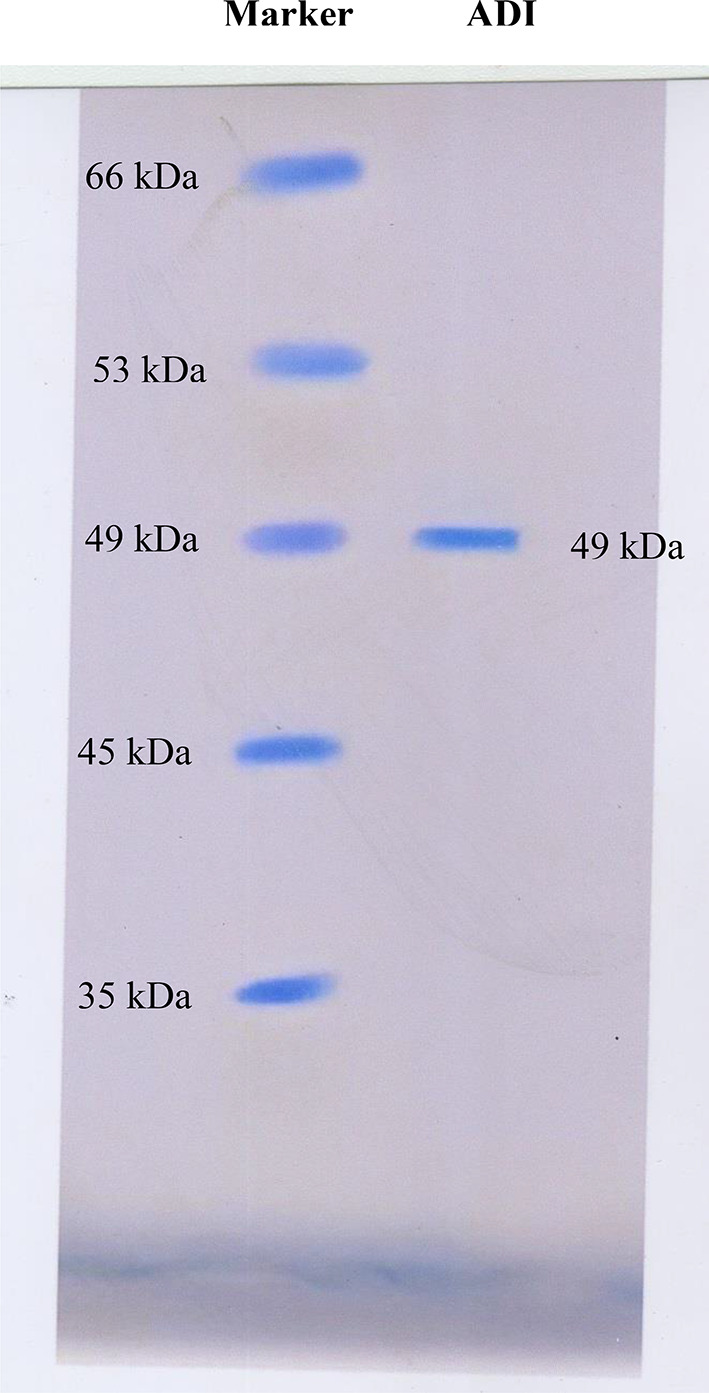
SDS-PAGE of ADI from *P. chrysogenum*

ADI from *P. chrysogenum* was isolated and purified using 85% (NH_4_)_2_SO_4_ precipitation, DEAE-cellulose and Sephadex G_200_. The results of purification are shown in Table 1. These results reveal a successful purification of ADI with a final specific activity of 13.8 Umg^− 1^ protein and 17.2-fold. The Purify of ADI was confirmed by SDS-PAGE and showed one band with MW of 49 kDa (Fig. 1).

**Table 1:** Purification of ADI from *P. chrysogenum*

Purification step	Total protein (mg)	Total activity (U)	Specific activity (Umg^− 1^)	Yield (%)	Fold of purification
Crude extract	300	240	0.8	100	1.0
85% (NH_4_) _2_ SO_4_	55	35	0.6	14.6	0.8
DEAE-Cellulose	16	22	1.4	9.2	1.7
Sephadex G-200	0.3	15	50.0	6.3	62.5

### Effect of L-arginine concentration on ADI activity

**Fig. 2:**
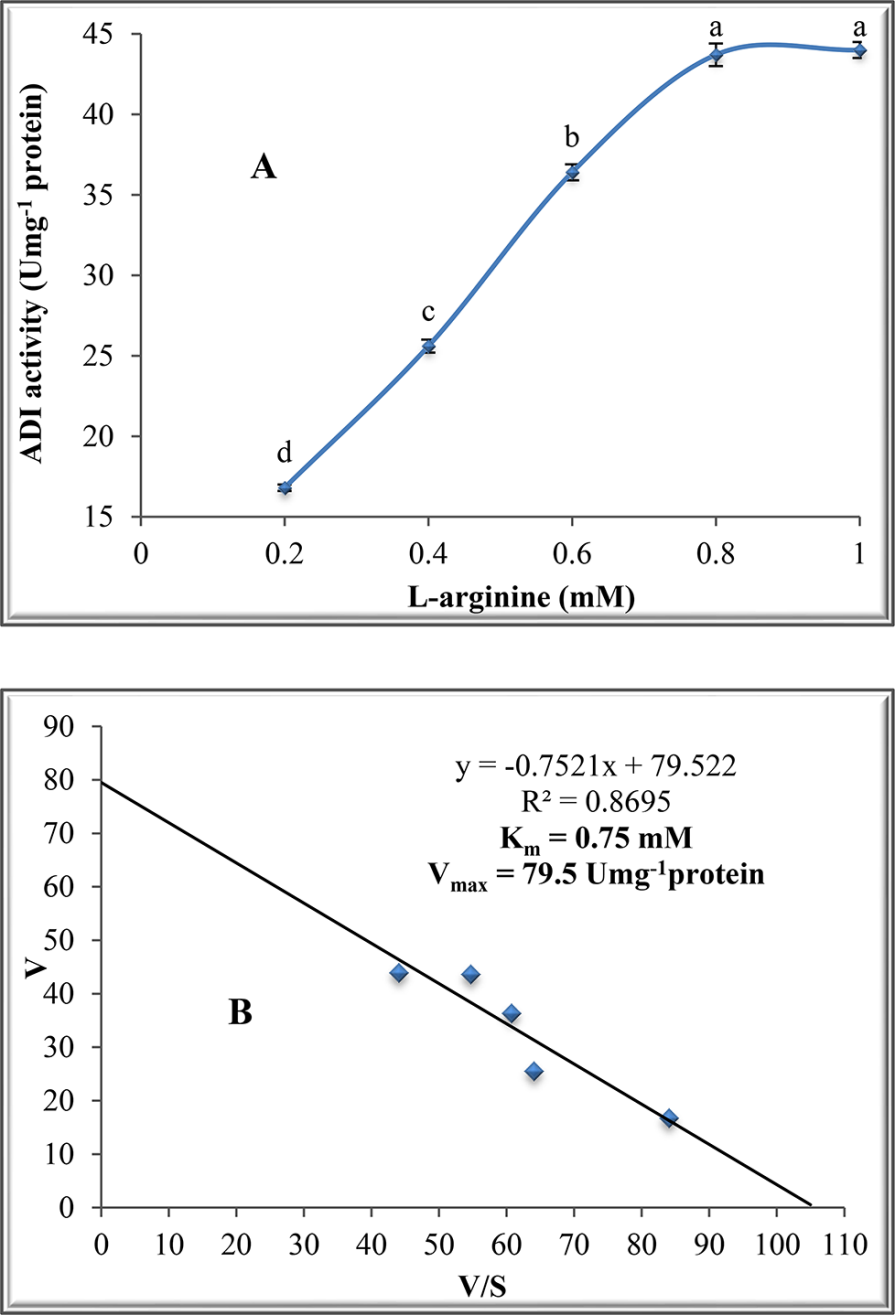
Effect of different substrate concentrations on ADI activity from *P. chrysogenum*. (**A)**: L-arginine as substrate and (**B)**: Eadie-Hofstee plot

The results in Fig. 2A show that there was a continuous increase in ADI activity with increasing the L-arginine concentration reaching 43.7 Umg^− 1^ protein at 0.8 mM, then there was a steady increase at 1.0 mM.

### Determination of K_m_ and V_max_ of purified ADI

Both K_m_ and V_max_ of the purified enzyme were calculated by Eadie-Hofstee plot, the calculated K_m_ and V_max_ values were 0.75 mM and 79.5 Umg^− 1^ protein (Fig. 2B), respectively.

### Effect of pH on the activity of purified ADI

**Fig. 3:**
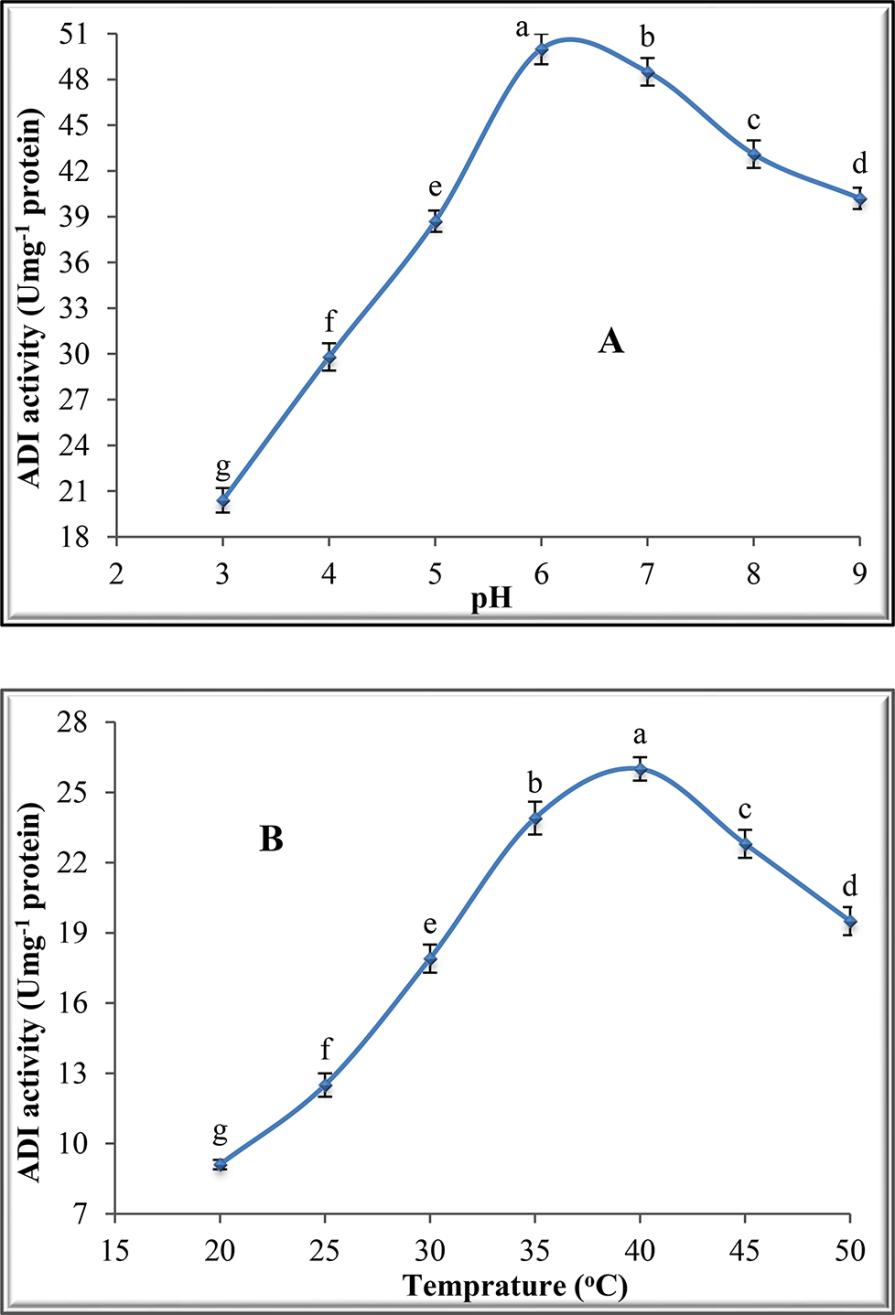
Effect of pH and temperature on purified ADI activity from *P. chrysogenum.* (**A)**: pH and (**B)**: Temperature

The effect of pH on ADI was investigated at pH range 3–9 (Fig. 3A). These results reveal a continuous increase in the enzyme activity from pH 3 to pH 6. After pH 6, any further increase in the pH reduced the enzyme activity, thus the optimal pH for ADI was 6.0.

### Effect of temperature on the activity of purified ADI

Studying the effect of temperature on ADI activity (Fig. 3B) indicates that there was a continuous increase in ADI activity with the increase in reaction temperature from 10^o^C to 40^o^C followed by gradual reduction with any further increase in temperature.

### Thermostability of the purified ADI

**Fig. 4:**
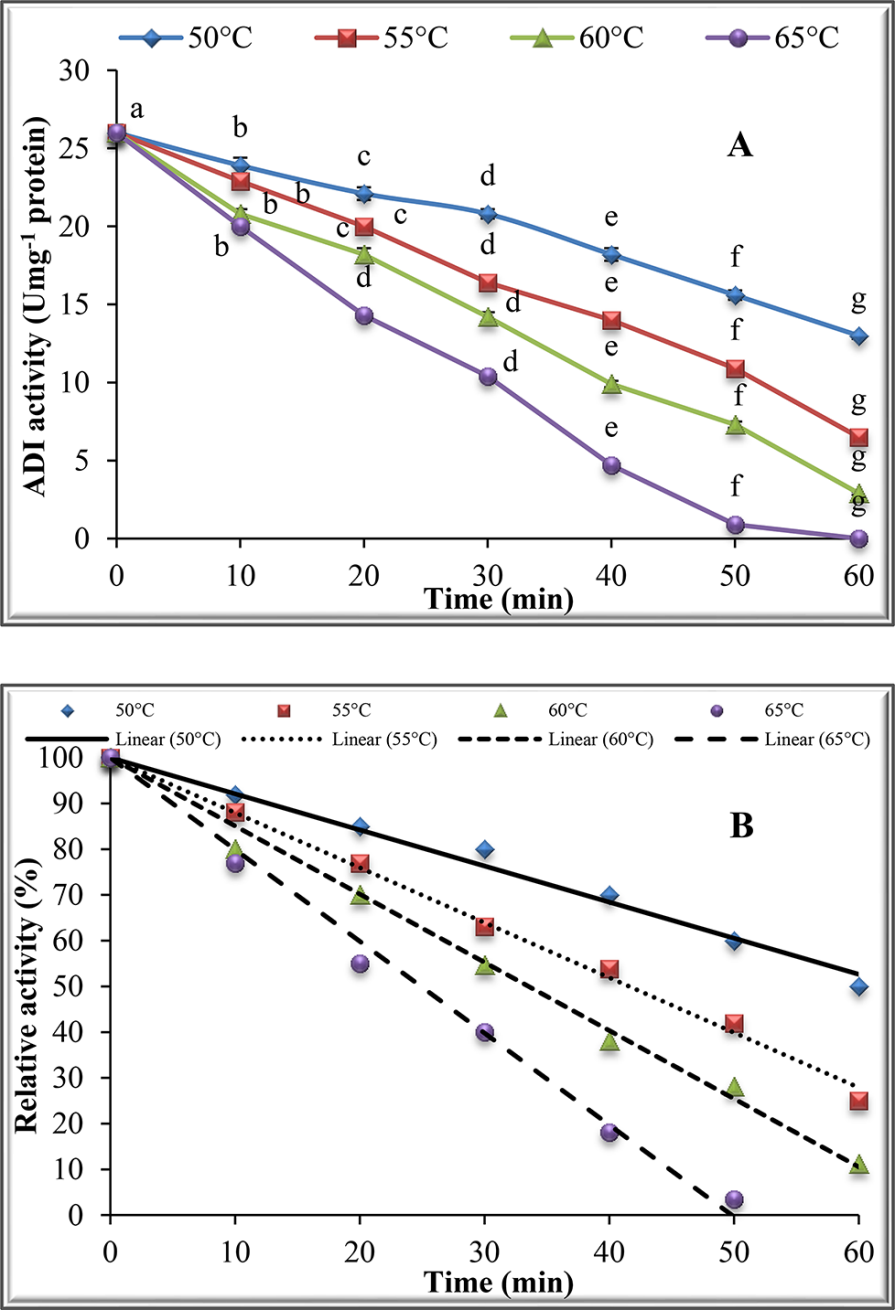
Thermostability of purified ADI from P. chrysogenum at 50 to 65oC.(**A**): enzyme activity and (**B**): relative activity

**Fig. 5:**
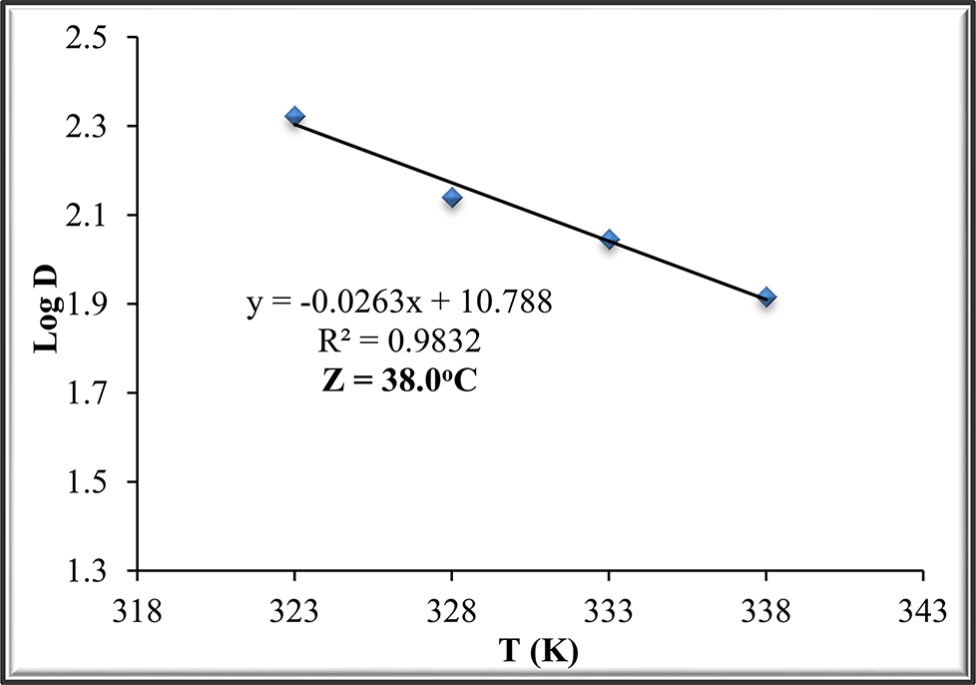
Determination of Z for purified ADI

The thermostability of ADI was investigated at 50, 55, 60 and 65^o^C. The results in Fig. 4A indicate a continuous decrease in ADI activity with increasing temperature and incubation time. The relation between the incubation time (min) and the relative activity of purified ADI is demonstrated in Fig. 4B. The results in Table 2 were calculated from Fig. 4B which show that t_0.5_ values of the enzyme were 63.37, 41.61, 33.50 and 24.86 min, respectively. However, K_d_ values of ADI were 0.0109, 0.0167, 0.0207 and 0.0279 min^− 1^, whereas the D-values were 210.5, 138.2, 111.3 and 82.6 min at the above-mentioned temperatures, respectively. Plotting the relation between T (K) against Log D straight lines (Fig. 5) was obtained for purified ADI and the calculated value of Z was 38.0^o^C.

**Table 2:** kinetic parameters calculated from Fig. 4

Temp.	Equation	R²	t_0.5_ (min)	K_d_ (min^− 1^)	D (min)
50	y = -0.789x + 100	0.9874	63.37	0.0109	210.5
55	y = -1.2017x + 100	0.9955	41.61	0.0167	138.2
60	y = -1.4925x + 100	0.9934	33.50	0.0207	111.3
65	y = -2.0109x + 100	0.993	24.86	0.0279	82.6

### Effect of adenosine compounds on ADI activity

**Fig. 6:**
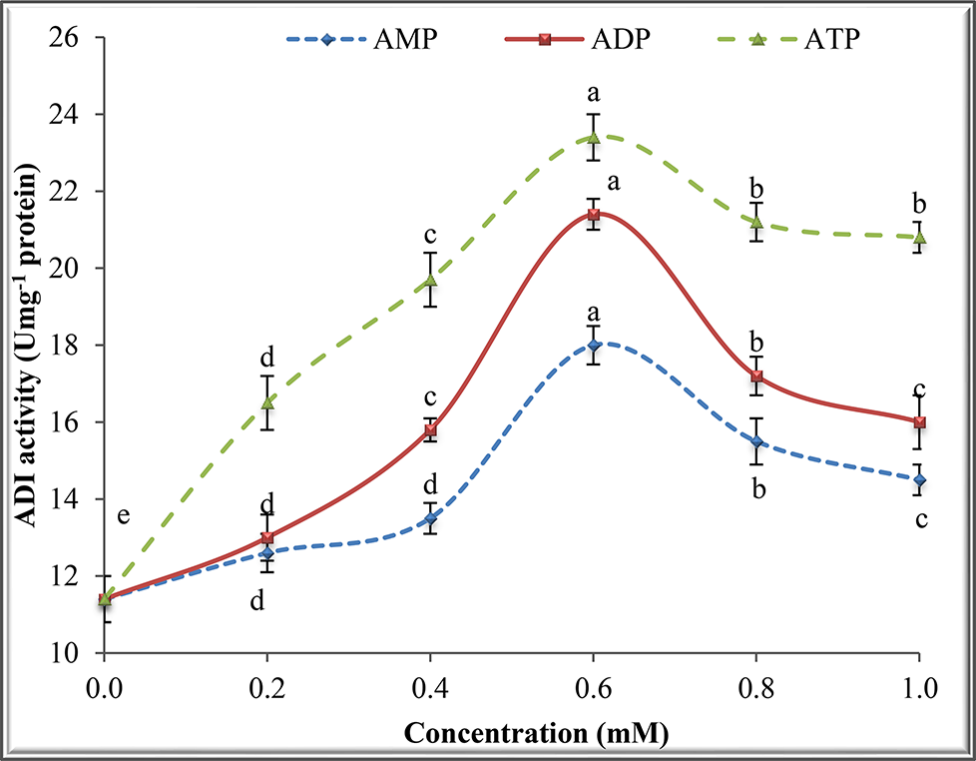
Effect of different concentrations of adenosine compounds on ADI activity from *P. chrysogenum*

The results in Fig. 6 show that the three adenosine compounds (ATP, ADP and AMP) activated ADI in a concentration-dependent manner. The optimal concentration of the three compounds was 0.6 mM after which the activity declined gradually at 0.8 and 1.0 mM. ATP was the most potent activator followed by ADP and later AMP.

### Effect of thiol compounds on ADI activity

**Fig. 7:**
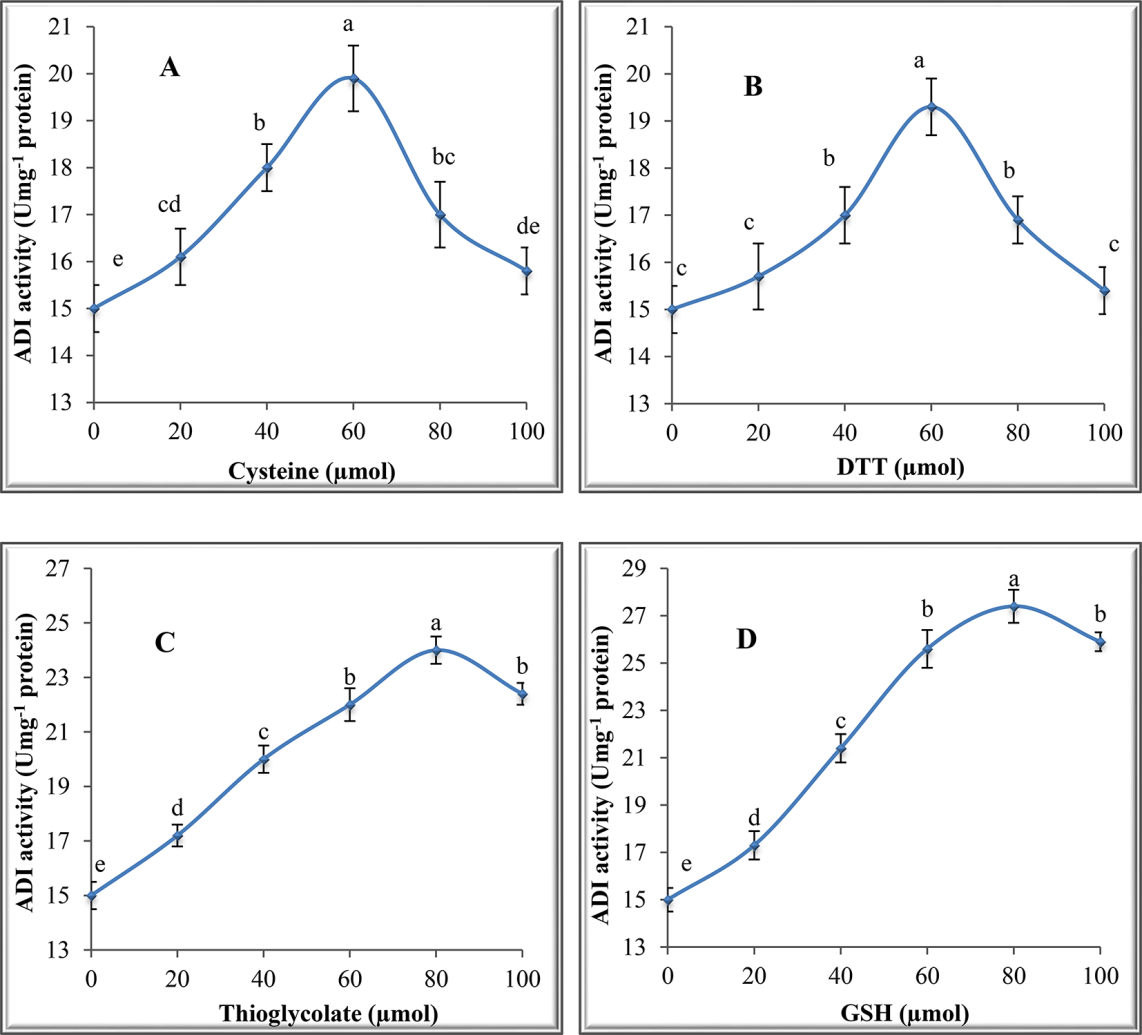
Effect of thiol compounds on ADI from *P. chrysogenum*. (**A)**: Cysteine, (**B)**: DTT, (**C)**: Thioglycolate and (**D)**: GSH

The results in Fig. 7A and B reveal a continuous increase in ADI activity with increasing cysteine and DTT concentration up to 60 µmol, where the enzyme activities were 19.9 and 19.3 units mg^− 1^protein for the two compounds, respectively. Continuous decline in ADI activity was recorded at 80 and 100 µmol of both compounds. The results in Fig. 7C and D demonstrate that 80 µmol was the optimal activating concentration for ADI by thioglycolate and GSH. However, at 100 µmol the activity declined and reached 22.4 and 25.9 units mg^− 1^ protein in the presence of each of the two compounds, respectively.

### Effect of chelating agents on ADI activity

**Fig. 8:**
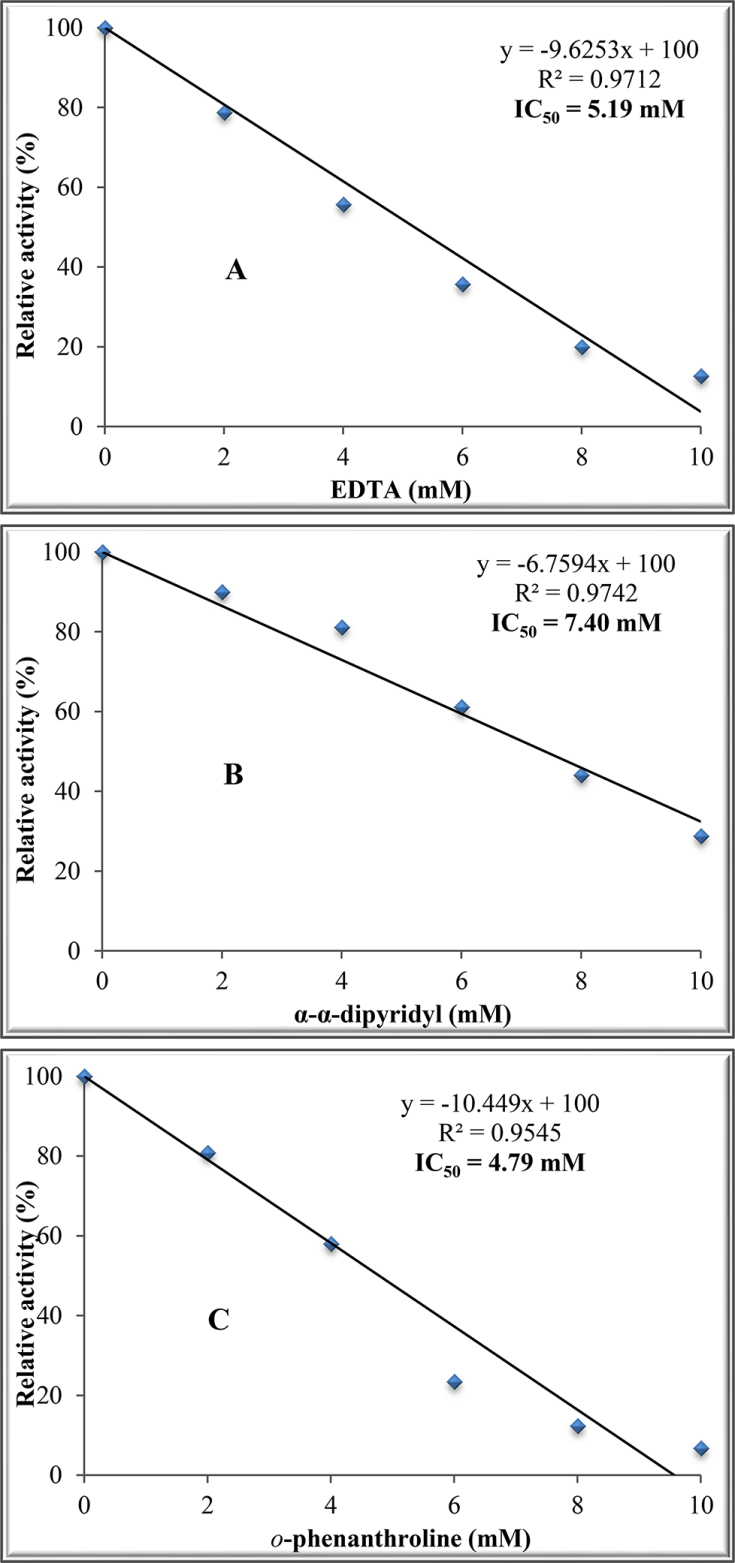
Effect of chelating agent compounds on ADI activity from *P. chrysogenum*. (**A)**: EDTA, (**B)**: α-α-dipyridyl and **(C)**: *o*-phenanthroline

The results in Fig. 8A, B and C indicate inhibition of purified ADI by EDTA, α-α-dipyridyl and *o*-phenanthroline, respectively in a concentration-dependent manner with IC_50_ of 5.19, 7.46 and 4.79 mM for the three compounds in the same order.

**Fig. 9:**
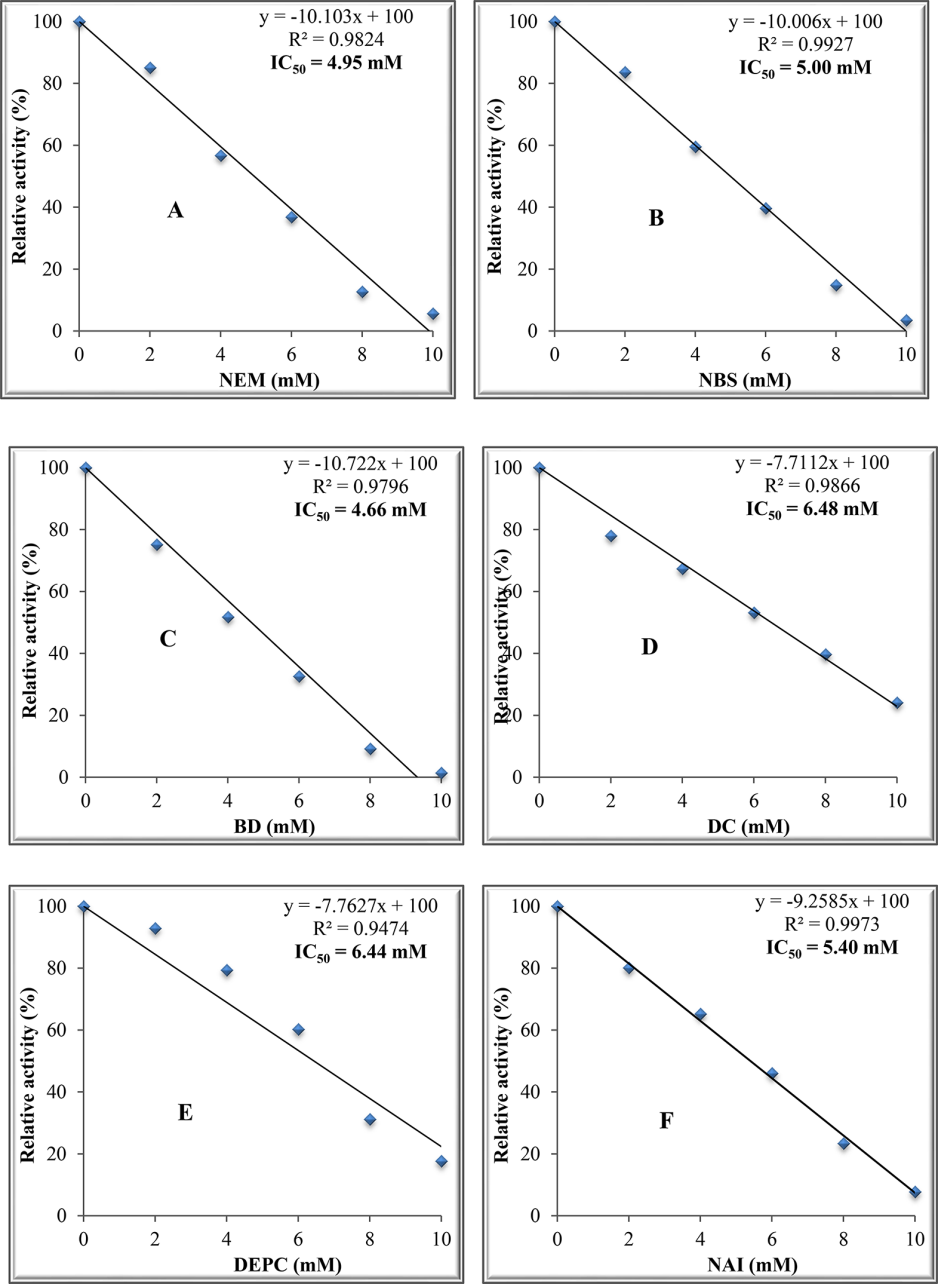
Effect of different modifiers on ADI activity from *P. chrysogenum*. (**A)**: NEM, (**B)**: NBS, (**C)**: BD, (**D)**: DC, **(E)**: DEPC and (**F)**: NAI

### Effect of various modifiers of the active groups on ADI activity

The results in Fig. 9A, B, C, D, E and F show inhibition of purified ADI by NEM, NBS, BD, DC, DEPC and NAI in a concentration-dependent manner. The IC_50_ values were 4.95, 5.0, 4.66, 6.48, 6.44 and 5.40 mM, respectively.

## Discussion

ADI was purified from *P. chrysogenum* by ammonium sulphate precipitation, DEAE-Cellulose and Sephadex G_200_. The final specific activity was 50.0 Umg^− 1^ protein which is higher than that reported for the enzyme from *A. nidulans* (12.9 Umg^− 1^ protein) [12] and *A. fumigatus* (26.7 Umg^− 1^ protein) [11]. Lower specific activity of 5.2 μm^− 1^ protein was found for ADI from *Enterococcus faecium* [36].

ADI was purified to homogeneity as shown in Fig.  61 where ADI is visible as single band of 49 kDa. Similar findings are reported for ADI from *Mycoplasma arthritidis* [37], *A. nidulans* (48 kDa) [12], and from thermophilic *A. fumigatus* (50 kDa) [11]. Lower molecular weights (45 kDa and 46.6 kDa) were reported for ADI from *Mycoplasma sarginini* and *Porphyromonas gingivalis* [38, 39], respectively. However, a higher molecular weight (186 kDa) for ADI was reported from *E. faecium* [36].

K_m_ value of ADI from *P. chrysogenum* was 0.75 mM. Higher K_m_ values of 8.8 and 4.8 mM were reported for ADI by [11, 12] for *A. fumigatus* and *A. nidulans*, respectively. The small value of K_m_ indicates a higher specificity of ADI towards L-arginine. The V_max_ value of ADI in the present work was 79.5 Umg^− 1^ protein, however, smaller V_max_ values of 28.7 and 59.5 Umg^− 1^ protein were reported for ADI from *A. fumigatus* and *A. nidulans*, respectively [11, 12].

The pH of ADI from *P. chrysogenum* was 6.0. ADI isolated from *Pseudomonas plecoglossicida* exhibited optimum pH at 6.5 [40]. ADI from *A. fumigatus* displayed maximum activity at a pH range from 5.5 to 8.0 [11]. The maximum activity of the *E. faecium* ADI enzyme was pH 7.0 [36]. However, ADI of *Enterococcus faecalis* [41] proved to be active over a wide range of pH values (4.0–10). ADI activity is dependent on the degree of ionization of some amino acid chains and pH profiles of the enzyme may suggest the identity of those groups [42]. The inhibitory effect of higher acidic or basic pH on ADI activity may indicate the change in the enzyme ionization state, modifying its surface, change and dissociation of subunits and consequently the disruption of the enzyme-substrate intermediate [43]. Extreme pH causes protein denaturation of ADI through different mechanisms. It can influence the ionization of the enzyme-substrate complex. Also, it can influence the ionization of the substrate and consequently affect the binding of the substrate to its enzyme. In addition, it can cause changes in protein structure which alert the stability of the enzyme. Furthermore, it can influence the ionization of various active groups of the enzyme molecule, thus it affects the affinity of the enzyme for its substrate [13].

The optimal temperature for ADI from *P. chrysogenum* was 40^o^C. The other reported optimal temperatures were 37^o^C for *Vibrio alginolyticus* [44] and 57^o^C for *A. fumigatus* [11]. In this investigation, the thermostability of ADI at temperature rates 50^o^C- to 65^o^C revealed a reduction of ADI activity with increasing the incubation time at various temperatures. The inactivation of ADI at high temperatures over the optimum may be due to hydrolysis of the peptide chain, aggregation, incorrect confirmation or destruction of amino acids [45]. At high temperatures, ADI is possibly inactivated by accumulation at hydrophobic locations which are displayed on denaturation [46]. The exact mechanism of thermal inactivation of enzyme protein is still uncertain. The first stage in thermal inactivation is the partial unfolding of the enzyme molecule. Under normal conditions, the catalytically active structure of the enzyme is kept by the balance between different monovalent ionic forces including hydrogen and hydrophobic interaction [1]. The active sites of the enzyme usually consist of numerous amino acid groups brought together only in the native three-dimensional structure of the enzyme. This unfolding develops the disassembling of the active center and thus the enzyme is inactivated [13]. The events occurring during thermal inactivation could be classified into two groups: (i) covalent changes include hydrolytic scission of disulphide of the enzyme protein, reduction of disulphide bonds (SــS) and oxidation of methionyl group. (ii) non-covalent changes in which the thermal unfolded enzyme molecules can transform [1].

The remaining activities of ADI from the thermal stability assay were plotted in the first-order enzyme inactivation rate graph. The slope from plotting relative activity (%) against time is expressed as half-life (t_0.5_). The half-life (t_0.5_) is the time needed for the enzyme to decompose the substrate until the enzyme loses half of its activity [47]. Another definition of half-life (t_0.5_) is the time required to reduce 50% of the initial enzyme activity at a particular temperature [28]. Deactivation is known to be a process where the secondary, tertiary or quaternary structure of a protein changes without breaking any covalent bonds [30]. The half-life (t_0.5_) was used to calculate thermal inactivation rate constant (K_d_) [48]. The deactivation of the enzyme is commonly expressed in terms of the values for the two parameters D and Z [49]. The D or decimal reduction value (in min) is described as the time the enzyme must be pre-incubated at a given temperature to maintain 10% residual activity [30]. Another definition of the D-value (decimal reduction time) is defined as the time needed for a 90% reduction in the initial activity and was calculated [29].

The inactivation rate was accelerated with the higher heating temperatures, while t_0.5_ and D values were reduced with increasing temperature, indicating a faster inactivation at higher temperatures. These variations can be due to numerous factors such as the pH and composition of the buffer during the thermal inactivation [28]. The dependence of the D-value on the temperature is expressed by the Z-value. The Z-value was derived from log D-values versus temperature. The Z-value indicates how many degrees of the temperature are required for decimal reduction time to be tenfold higher or lower [28].

ATP, ADP and AMP activated ADI and this indicates that the reaction of ADI is exothermic. Adenosine compounds activated other microbial enzymes such as protease [50]. The four thiol compounds cysteine, DTT, thioglycolate and GSH activated ADI from *P. chrysogenum* with different levels. DTT was reported to protect the loss of enzyme activity which occurs by the oxidation of sulfhydryl groups during incubation time [31]. Also, this thiol compound may cause a lowering of the K_m_ of the enzyme to its substrate thereby activating the enzyme. El-Shora et al. [51] reported the enhancement of bacterial cholesterol oxidase by DTT, cysteine and GSH. DTT activated fungal *β*-glucanase by offering protection against the oxidation of sulfhydryl groups [52] and cysteine activated other microbial enzymes such as asparaginase [53].

The chelating agents EDTA, α-α-dipyridyl and *o*-phenanthroline inhibited ADI in a concentration-dependent manner indicating that ADI from *P. chrysogenum* is metallo-enzyme. Other microbial enzymes have been reported to be inhibited by EDTA such as *β*-lactamase [54], protease [31] and *β*-glucanase [52].

The present work shows the inhibition of ADI activity by NEM, NBS, BD, DC, DEPC and NAI indicating the essentiality of sulfhydryl, tryptophanyl, arginyl, lysyl, histidyl and tyrosyl groups, respectively for enzyme catalysis. In support, Jiang et al. [55] reported the necessity of the tested active groups for ADI catalysis. Also, Lu et al. [56] found the importance of the cysteinyl group for ADI performance. Generally, it is remarkable that investigation of the chemical modification of the enzyme could selectively target the enzyme groups specifically at the active site. These results reveal the usefulness of such a study in the preliminary identification of the groups taking part in the enzyme catalysis.

## Conclusion

*P. chrysogenum* possessed arginine metabolizing capacity and ADI was purified to homogeneity. This is the first report on the purification of ADI from *P. chrysogenum*. The purified ADI from *P. chrysogenum* has several beneficial properties needed for a therapeutic and industrial enzyme. It has a wide range of activity and stability at various temperatures. The substrate specificity towards L-arginine is also very high which means that it could be used in low amounts to achieve the desired effect.

## Data Availability

The data sets used and analyzed during the current study are available from the authors upon reasonable request.
